# Dysfunctional effort-based decision-making underlies apathy in genetic cerebral small vessel disease

**DOI:** 10.1093/brain/awy257

**Published:** 2018-10-20

**Authors:** Campbell Le Heron, Sanjay Manohar, Olivia Plant, Kinan Muhammed, Ludovica Griffanti, Andrea Nemeth, Gwenaëlle Douaud, Hugh S Markus, Masud Husain

**Affiliations:** 1 Nuffield Department of Clinical Neurosciences, University of Oxford, Oxford, UK; 2 Department of Experimental Psychology, University of Oxford, Oxford, UK; 3 New Zealand Brain Research Institute, Christchurch, New Zealand; 4 Division of Clinical Neurology, John Radcliffe Hospital, Oxford University Hospitals Trust, Oxford, UK; 5 Wellcome Centre for Integrative Neuroimaging, University of Oxford, Oxford, UK; 6 Stroke Research Group, Department of Clinical Neurosciences, University of Cambridge, Cambridge, UK

**Keywords:** apathy, decision-making, reward, CADASIL, small vessel cerebrovascular disease

## Abstract

Apathy is a syndrome of reduced motivation that commonly occurs in patients with cerebral small vessel disease, including those with the early onset form, CADASIL (cerebral autosomal dominant arteriopathy with subcortical infarcts and leukoencephalopathy). The cognitive mechanisms underlying apathy are poorly understood and treatment options are limited. We hypothesized that disrupted effort-based decision-making, the cognitive process by which potential rewards and the effort cost required to obtain them is integrated to drive behaviour, might underlie the apathetic syndrome. Nineteen patients with a genetic diagnosis of CADASIL, as a model of ‘pure’ vascular cognitive impairment, and 19 matched controls were assessed using two different behavioural paradigms and MRI. On a decision-making task, participants decided whether to accept or reject sequential offers of monetary reward in return for exerting physical effort via handheld dynamometers. Six levels of reward and six levels of effort were manipulated independently so offers spanned the full range of possible combinations. Choice, decision time and force metrics were recorded. Each participant’s effort and reward sensitivity was estimated using a computational model of choice. On a separate eye movement paradigm, physiological reward sensitivity was indexed by measuring pupillary dilatation to increasing monetary incentives. This metric was related to apathy status and compared to the behavioural metric of reward sensitivity on the decision-making task. Finally, high quality diffusion imaging and tract-based spatial statistics were used to determine whether tracts linking brain regions implicated in effort-based decision-making were disrupted in apathetic patients. Overall, apathetic patients with CADASIL rejected significantly more offers on the decision-making task, due to reduced reward sensitivity rather than effort hypersensitivity. Apathy was also associated with blunted pupillary responses to incentives. Furthermore, these independent behavioural and physiological markers of reward sensitivity were significantly correlated. Non-apathetic patients with CADASIL did not differ from controls on either task, whilst actual motor performance of apathetic patients in both tasks was also normal. Apathy was specifically associated with reduced fractional anisotropy within tracts connecting regions previously associated with effort-based decision-making. These findings demonstrate behavioural, physiological and anatomical evidence that dysfunctional effort-based decision-making underlies apathy in patients with CADASIL, a model disorder for sporadic small vessel disease. Reduced incentivization by rewards rather than hypersensitivity to effort costs drives this altered pattern of behaviour. The study provides empirical evidence of a cognitive mechanism for apathy in cerebral small vessel disease, and identifies a promising therapeutic target for interventions to improve this debilitating condition.

## Teaser

Le Heron *et al*. demonstrate, using behavioural, physiological and imaging techniques, converging evidence that reduced reward sensitivity underlies apathy in patients with a monogenic form of cerebral small vessel disease. This specific change in effort-based decision making points to potential treatment avenues for apathy.

## Introduction

Apathy, a disorder of motivation that manifests as a reduction in goal-directed behaviour, is an increasingly recognized complication of sporadic cerebral small vessel disease (SVD) (van der Mast *et al.*, 2008; Hollocks *et al.*, 2015; Lohner *et al.*, 2017). Apathy is also a cardinal feature of cerebral autosomal dominant arteriopathy with subcortical infarcts and leukoencephalopathy (CADASIL), a monogenic form of SVD caused by mutations within the *NOTCH3* gene and characterized by extensive damage to white matter brain regions (Chabriat *et al.*, 2009; Reyes *et al.*, 2009). It occurs in at least 40% of patients with CADASIL (Reyes *et al.*, 2009). Furthermore, the presence of SVD may also modulate the development of apathy in other neurological conditions including Alzheimer’s disease and stroke (Lavretsky *et al.*, 2008; Starkstein *et al.*, 2009; Jonsson *et al.*, 2010; Brodaty *et al.*, 2013). However, the cognitive mechanisms underlying apathy remain poorly understood, despite increased recognition of its prevalence in many neurological and psychiatric conditions and its association with reduced quality of life and poor health outcomes (Lyketsos *et al.*, 2002; Aarsland *et al.*, 2009; Mayo *et al.*, 2009; Treadway and Zald, 2011; Caeiro *et al.*, 2012; Hollocks *et al.*, 2015; Muhammed and Husain, 2016).

Prominent theories of apathy have considered it as a disorder of goal-directed behaviour (Marin, 1991; Levy and Dubois, 2006). However, goal-directed behaviour is itself a complex construct, relying on multiple cognitive processes (Balleine and O’Doherty, 2010). Although this general concept has proved useful, a mechanistic explanation for why goal-directed behaviour is reduced in apathy, based on dysfunction of specific cognitive processes, has remained elusive. Effort-based decision-making is a cognitive process that lies at the heart of normal goal-directed behaviour (Kurniawan *et al.*, 2011; Rushworth *et al.*, 2011). A recent framework has suggested dysfunction of this process as a possible cognitive mechanism underlying apathy (Le Heron *et al.*, 2017). Effort-based decision-making describes the process of integrating information about the costs of actions and potential rewards resulting from them, in deciding whether a behaviour is worth performing (Kurniawan *et al.*, 2011). It is proposed that such evaluations are crucial to understanding individual differences between how people act in daily life. For example, whether it is worth cleaning a house or mowing the lawn varies depending upon how individuals weigh up the costs of performing these actions compared to the potential rewards associated with these behaviours. Many studies have now demonstrated that such costs, rewards, and an integrated value signal (rewards – costs) are encoded neurally to drive goal-directed behaviour (Prevost *et al.*, 2010; Rushworth *et al.*, 2012).

Intriguingly the neural regions that subserve effort-based decision-making, including anterior cingulate cortex (ACC), orbitofrontal cortex (OFC) and ventral striatum (Croxson *et al.*, 2009; Prevost *et al.*, 2010; Schmidt *et al.*, 2012; Klein-Flugge *et al.*, 2016; Chong *et al.*, 2017; Hauser *et al.*, 2017), are the same regions that have been associated with apathy in neuroimaging studies across different modalities and neurological conditions (Kos *et al.*, 2016; Le Heron *et al.*, 2017). Furthermore, animal models of effort-based decision-making have causally linked damage to both ACC and ventral striatum with behavioural changes that resemble the clinical phenotype of apathy. Lesions to these regions induce a seemingly apathetic state in animals, in which they are no longer as willing to invest effort for reward (Salamone *et al.*, 1994; Walton *et al.*, 2002; Hauber and Sommer, 2009). Thus, disrupted effort-based decision-making provides a plausible cognitive mechanism for the reduced goal-directed behaviour that defines apathy. Such a disruption could result from reduced sensitivity to rewarding outcomes, increased sensitivity to effort costs, or a global reduction in willingness to engage, but which (if any) of these factors is related to apathy remains unknown.

Behavioural evidence for disruption of effort-based decision-making associated with apathy (Le Heron *et al.*, 2018) or changes in motivation (Le Bouc *et al.*, 2016) has recently been reported in Parkinson’s disease. Disrupted physiological responses to incentives have also been associated with apathy in this condition (Lawrence *et al.*, 2011; Martinez-Horta *et al.*, 2014; Muhammed *et al.*, 2016). However, no mechanistic studies have been carried out in SVD—whether sporadic or genetic—and it is unclear whether the results from patients with Parkinson’s disease would generalize to a very different clinical group such as patients with SVD. Similarly, there is also a dearth of imaging studies of apathy in SVD compared to other neurodegenerative conditions, such as Parkinson’s disease (Wen *et al.*, 2016) or Alzheimer’s disease (Theleritis *et al.*, 2014). To date, there is only one report of the neural correlates of apathy in CADASIL (Jouvent *et al.*, 2011), and, as far as we are aware, there is no previous report that has examined associations with specific changes in white matter tracts, despite this being the pathognomonic site of pathology in the disorder (Chabriat *et al.*, 2009).

Apathy in sporadic SVD has also been associated with global burden of vascular disease; however, these whole-brain measures do not allow for a mechanistic interpretation of underlying causes, nor account for potential confounding effects of co-existent neurodegenerative pathology (Lavretsky *et al.*, 2008; Jonsson *et al.*, 2010; Kim *et al.*, 2013; Attems and Jellinger, 2014; Hollocks *et al.*, 2015). One recent study has examined the relationship between fractional anisotropy (a measure of white matter tract integrity) and apathy in sporadic SVD, reporting reductions within the anterior cingulum, uncinate fasciculus and fornix associated with apathy (Hollocks *et al.*, 2015). These white matter tracts link brain regions strongly implicated in the development of apathy in other conditions—regions in which the process of effort-based decision-making is instantiated (Le Heron *et al.*, 2017). However, no behavioural measures of decision-making were available in these patients. Overall, empirical evidence for disrupted effort-based decision-making is lacking in SVD, and only sparse in the general apathy literature. Furthermore, to the best of our knowledge, no study in any neurological condition has examined behavioural, physiological and anatomical components of effort-based decision-making in the same group of apathetic patients, limiting the strength of conclusions that can be drawn from the existing body of work.

Sporadic SVD is a significant public health burden and is the most common cause of vascular cognitive impairment and vascular dementia (Pantoni, 2010). However, it occurs in a predominantly elderly population in whom co-existent neurodegenerative pathology is often present, limiting the study of underlying neurobiological mechanisms (Medical Research Council Cognitive Function and Aging Study, 2001; Attems and Jellinger, 2014). CADASIL has very similar pathology and pattern of cognitive impairment to sporadic SVD, but without the co-existent neurodegenerative changes or significant co-morbidities due to the younger age of patients (Charlton *et al.*, 2006; Chabriat *et al.*, 2009). It therefore provides a model of pure SVD and vascular cognitive impairment in which to study mechanistic questions (Charlton *et al.*, 2006; Duering *et al.*, 2013).

Here, we examined reward and effort processing in patients with CADASIL, with and without clinical apathy, as well as matched healthy controls. We used behavioural, physiological and anatomical techniques in the same participants to investigate whether disrupted effort-based decision-making underlies apathy in this condition, and specifically whether alterations in this process were associated with reward insensitivity, effort hypersensitivity or global changes in willingness to engage. Participants completed an effort-based decision-making task in which they weighed up offers of monetary reward in return for exerting physical effort. Computational analysis of these choices provided an estimate of each person’s reward and effort sensitivity. This allowed us to determine if any observed changes in decision-making associated with apathy were specific to reward or effort processing. Additionally, a physiological marker of reward sensitivity—the degree of pupillary dilatation to reward—was determined using a previously validated eye movement task (Muhammed *et al.*, 2016). The noradrenergic system is considered to mediate preparation of effort (Varazzani *et al.*, 2015), and thus pupillary dilatation in response to rewards may provide an autonomic marker of motivation (Chiew and Braver, 2014; Manohar and Husain, 2015). This metric was compared between apathy and no-apathy groups, and to each person’s behaviourally derived reward sensitivity. Finally, using a tract-based spatial statistics (TBSS) analysis of diffusion imaging sequences (Smith *et al.*, 2006), we assessed whether apathy is associated with disruption of tracts linking neural regions crucial for effort-based decision-making. Thus, we have used a multimodal approach to investigate whether specific changes in the process of effort-based decision-making underlie the clinical phenotype of apathy in patients with CADASIL, a model of pure SVD.

## Materials and methods

### Ethics

The study was approved by the local ethics committee and written consent was obtained from all subjects in accordance with the Declaration of Helsinki.

### Participants

Nineteen patients with a genetically confirmed diagnosis of CADASIL, all with cysteine changing *NOTCH3* mutations, were recruited from two regional specialist centres (in Cambridge and Oxford, UK). Exclusion criteria included physical disability such that a patient was unable to squeeze a hand-held dynamometer or previously documented large vessel stroke. Three patients with a history of lacunar stroke were included because this is an intrinsic feature of CADASIL, and is also a cardinal feature of sporadic SVD (Chabriat *et al.*, 2009; Pantoni, 2010). Nineteen healthy age and gender matched controls were recruited via a local database.

All participants completed the behavioural and eye-tracking experiments. One patient with CADASIL did not undertake an MRI scan due to severe claustrophobia. Additionally, two further patients were excluded following image acquisition, one because of significant movement artefact and the other due to an acute clinical event occurring between the behavioural testing and the time of imaging (which had been delayed by 1 month). Demographics are presented in Table 1.

**Table 1:** Demographics

**Measure**	**Control *n = *19**	**CADASIL *n = *19**	***P*-value**	**CADASIL No apathy *n = *8**	**CADASIL Apathy *n* = 11**	***P*-value**
Age	54.5 ± 11.2	54.3 ± 10.3	0.96	55.8 ± 11.1	53.3 ± 10.1	0.62
Gender, female/male	12/7	13/6	0.73	6/2	7/4	0.6*
Apathy, LARS, range −36 to 36	−28 ± 3.6	−20 ± 11.8	**0.007**	−29 ± 3.5	−13.5 ± 11.4	**0.0018**
Apathy, AES, range 0−72	30.8 ± 10.3	34.6 ± 10.4	0.27	26.9 ± 5.7	40.2 ± 9.6	**0.0028**
GDS-total^a^, range 0−15	1 ± 1.8	5.7 ± 5.0	**<0.0001**	2.1 ± 2.5	8.5 ± 4.6	**0.003**
GDS-apathy, range 0−3	0.5 ± 0.9	1.2 ± 1.1	**0.04**	0.25 ± .046	1.9 ± 0.87	**<0.0001**
GDS-depression, 0−12	0.7 ± 1.4	4.5 ± 4.2	**<0.0001**	1.9 ± 2.1	6.6 ± 4.3	**0.012**
Quality of life: CANTRIL, range 0−10	8.1 ± 1.6	6.6 ± 2.2	**0.017**	8 ± 0.9	5.5 ± 2.3	**0.010**
Quality of life: WHO5, range 0−25	N/A	13.8 ± 6.1	N/A	18.1 ± 3.5	10.6 ± 5.7	**0.0005**

Values in bold represent *P* < 0.05. AES = Apathy Evaluation Scale; GDS = Geriatric Depression Scale; LARS = Lille Apathy Rating Scale.

*Chi-square test.

^a^Data for the GDS was missing for one participant.

### Disease, cognitive and questionnaire measures

Apathy was assessed using the Lille Apathy Rating Scale (LARS) (Sockeel *et al.*, 2006), and the Apathy Evaluation Scale (AES) self-report version (Marin *et al.*, 1991; Clarke *et al.*, 2007), which were both systematically administered to improve the sensitivity of the apathy diagnosis. Apathy was diagnosed if either the LARS score was >−22 or the AES score was >37 (equivalent to at least mild-moderate apathy). Physical signs were assessed using the National Institutes of Health Stroke Scale (Lyden, 2017). Baseline cognitive levels were formally assessed using the Addenbrookes’s Cognitive Examination III (ACE-III, Hsieh *et al.*, 2013). Given previous work suggesting cognitive deficits in CADASIL particularly involve executive functions (Charlton *et al.*, 2006), a digit span working memory task (Groth-Marnat, 2000), the Trail-Making Task parts A and B (Tombaugh, 2004), and a design fluency task in which participants were required to generate as many novel designs by connecting up to five dots with straight lines in a 2-min period (Tucha *et al.*, 2012) were also administered. Depressive symptoms were assessed using the Geriatric Depression Scale (GDS), which has been the most extensive index used in SVD apathy research (van der Mast *et al.*, 2008; Ligthart *et al.*, 2012; Hollocks *et al.*, 2015). Quality of life was assessed using a Cantril Ladder (Cantril, 1965), in which participants rated their overall current quality of life on a visual scale ranging from 1 to 10, and the WHO-5 Well-Being Index (Topp *et al.*, 2015).

### Effort-based decision-making paradigm

**Figure 1:**
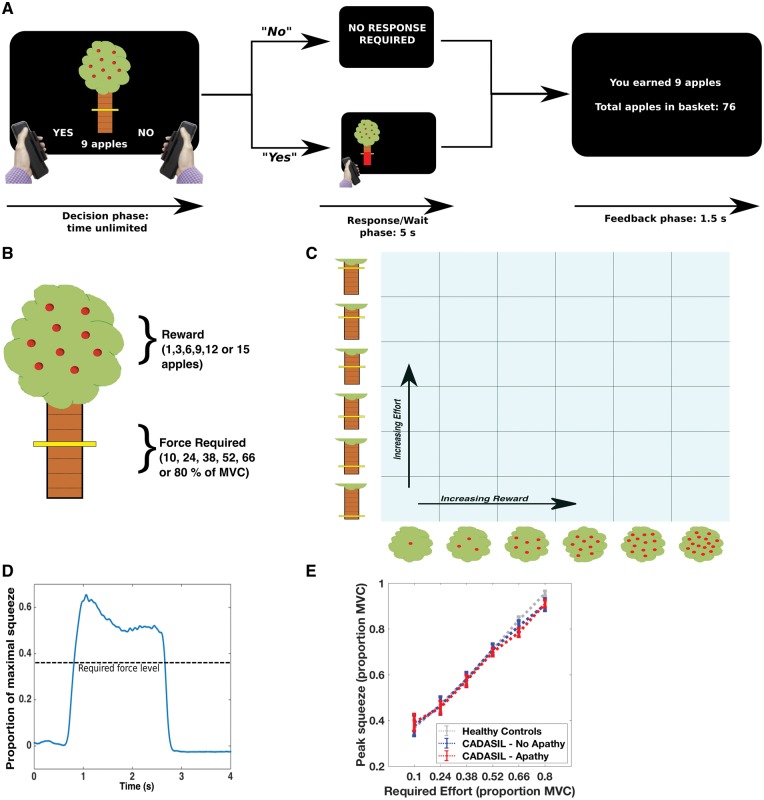
**Effort-based decision-making task.** (**A** and **B**) On a trial-by-trial basis participants were presented with offers of reward (apples on an apple tree, with each apple worth 1p) in return for exerting physical effort, ranging between 10% and 80% of a subject’s previously determined maximal voluntary contraction (MVC). If they accepted an offer (by squeezing the left-hand grip) the tree moved to the left or right of the screen, indicating which hand they had to respond with. They had a 5-s window within which to achieve the required force level. If they rejected the offer (by squeezing the right-hand grip) they waited the same 5-s period. (**C**) Participants worked through 180 trials, which pseudo-randomly, evenly sampled the 6 × 6 decision space over five blocks. (**D**) Example force trace from a single trial. (**E**) All groups (healthy controls, CADASIL no apathy and CADASIL apathy) modulated their force output to task requirements: mean ± standard error (SE).

Participants were administered an effort-based decision-making task on a desktop computer running Pyschtoolbox (psychotoolbox.org) implemented within MATLAB (MathWorks, USA). They made sequential decisions of whether to accept an offer of reward in return for exerting effort via individually calibrated handheld dynamometers (SS25LA, BIOPAC Systems; Fig. 1A). Each offer was presented on the screen as a cartoon apple tree. Reward for the current trial was indicated by the number of apples on the tree (1, 3, 6, 9, 12 or 15) and numerically displayed underneath the tree. Each apple was worth 1p. Effort required to obtain the reward was indicated by the height of the yellow bar on the tree trunk, with the six possible levels corresponding to 10, 24, 38, 52, 66 and 80% of a participant’s maximal voluntary contraction (Fig. 1B). The six reward and six effort levels were systematically combined and the resultant 36 conditions were sampled evenly in a pseudo-randomized order across five blocks, for a total of 180 trials (Fig. 1C). This meant all participants received the same offers, presented in the same order.

Participants were instructed to weigh up the effort costs against the reward on offer for each trial, and decide ‘If it is worth it—is it worth squeezing that hard for that number of apples?’ If they accepted an offer (by exerting a small squeeze on the left hand-grip) they had to squeeze to the required force and hold above this level for at least 1 s within a 5-s response window, after which they were ‘rewarded’ with the apples on offer. During the squeeze, online force feedback was shown as a red bar that indicated current force relative to the target line. Conversely, if participants rejected an offer (by exerting a small squeeze on the right-hand grip) they waited an equivalent time (to control for temporal discounting effects) before moving onto the next offer. Therefore, on each trial participants decided whether the value of an offer was worth engaging with, compared to doing nothing for the equivalent time (Fig. 1A).

Before starting the experiment, participants practised each force level with each hand to familiarize themselves with the effort required, and completed a practice block in which they made decisions on the full range of options in the experiment. We included a number of features to reduce potential effects of fatigue on choice, including varying response side (signalled after an offer was accepted), only requiring subjects to squeeze on 75% of accepted trials and allowing subjects to rest between each block of 36 trials. In addition to choice (accept/reject), decision latency and force metrics (sampled at 500 Hz) were recorded for each trial (Fig. 1D and E; see Supplementary material for further details).

### Eye movement paradigm

**Figure 2:**
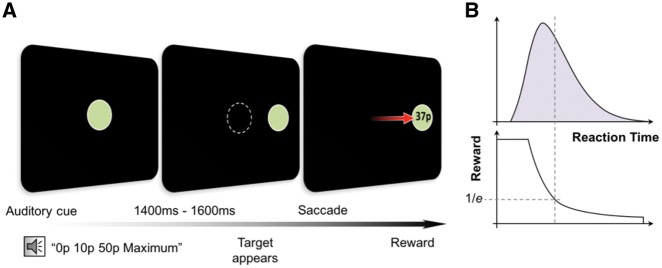
**Eye movement task.** (**A**) Participants fixated on a disc displayed on a desktop monitor linked to an infrared eye tracker. At the beginning of each trial participants heard an auditory cue (‘0p/10p/50p maximum’) informing them the maximal amount they could win on that trial. Following a delay of 1400–1600 ms the central disc disappeared and concurrently a target disc appeared on one side, cueing a saccade to this location. Participants were rewarded with a proportion of the maximum amount available based on the time taken to reach the target. (**B**) Although the absolute amount of reward varied with reaction time, this was dynamically adjusted according to the mean reaction time of the last 20 trials. Therefore, difficulty level was maintained across the experiment and overall participants received equal reward amounts. Adapted from Muhammed *et al.* (2016) with permission.

The eye movement task has been described in detail in a previous publication (Muhammed *et al.*, 2016 and Supplementary material). Briefly, participants performed saccadic eye movements from a central fixation point to a peripheral target to earn monetary rewards, while pupil diameter and eye position were monitored using an infrared eye tracker (Eyelink 1000, SR Research; Fig. 2). They were seated 60 cm from a 21-inch CRT (1024 × 768 pixels; 100 Hz refresh rate) in a dimly lit room. Three levels of reward were used: 0p, 10p and 50p; representing the maximal amount available on that trial. Participants were informed of reward level on the current trial at the beginning via an audio cue whilst they maintained central fixation. Following a period of 1400–1600 ms, the central fixation point was extinguished and a peripheral target displayed, signalling participants to saccade to this target. Reward earned (as a proportion of the maximum available for that trial) depended on time to reach this target; however, this amount was dynamically adjusted dependent on performance over the preceding 20 trials. This maintained a consistent difficulty level across the experiment, accounting for potential confounding factors such as fatigue and baseline reaction time and ensuring equal rewards were earned by all participants. Participants performed five blocks of 54 trials each, with the three reward levels interleaved through each block. Disc luminance was matched across all trials so as not to affect pupil dilation. Baseline pupil size was recalculated at the beginning of each trial.

### Imaging protocol

Participants were scanned in a 3 T Siemens Verio scanner with a 12-channel head coil between January 2015 and June 2017. They were instructed to lie as still as possible. The neuroimaging protocol included diffusion-weighted (19:52 s), T_1_-weighted (4:54 s) and T_2_-FLAIR (4:32 s) sequences. Whole-brain diffusion weighted imaging was performed using an echo-planar sequence (repetition time = 8900 ms, echo time = 91.2 ms, voxel size = 2 mm isotropic, b-value = 1500 s/mm^2^). Images were obtained using diffusion-sensitizing gradients in 60 isotropically-distributed orientations optimized to evenly sample the whole diffusion sphere, acquired in opposite phase encoding directions (AP and PA) to allow for more robust distortion correction. 4 b = 0 images were obtained in each phase encoding direction.

### Statistical analysis

#### Decision-making task

Choice data were analysed in two separate ways. We used a repeated measures ANOVA to test whether the proportion of offers accepted in each of the 36 (reward × effort) conditions varied as a function of reward, effort, apathy or their interactions. The dependent variable was arcsine transformed because it did not meet assumptions of linearity and a Greenhouse-Geisser correction was applied because assumptions of sphericity were not met (Mauchly’s test: *ɛ* = 0.317, *P* < 001). Residuals were examined following the analysis to ensure an approximately normal distribution.

We also used a computational model of choice to estimate, for each participant, the degree that reward and effort changed the value of an offer. We selected the model based on a comparison of candidate models (identified from prior decision-making literature) (Prevost *et al.*, 2010; Chong *et al.*, 2017), using standard methods (minimization of Bayesian information criterion and visual inspection of individual and group model fits to raw data); (Supplementary material and Supplementary Fig. 1).

An exponential model closely approximated both individual and group average raw choices (Fig. 4B and C):

(1) $$Value\;=\;\alpha Reward\times e^{-\beta Effort}+k$$

Here *α* estimated the degree that reward (discounted by effort) increased the value of an offer, *β* estimated the degree that effort reduced the value of an offer, and *k* the baseline tendency to accept an offer (the value of accepting an offer if zero reward was available). *β* and *k* parameter estimates, and log(*α*), were normally distributed and therefore compared using unpaired *t*-tests.

Decision time was analysed by repeated measures ANOVA with decision-type (accept or reject) a within subject variable and group (CADASIL-apathy, CADASIL-no apathy or control) a between subject variable. Decision time also provides a metric of task engagement. We predicted regions of decision space where subjects were more variable in their choice (and therefore more uncertain) would have longer decision times than ‘easier’ choices (for example regions of decision space that are always accepted or rejected). To test whether decision time was modulated by decision difficulty we split each participant’s decisions into easy (accepted <25% or >75%) and hard (accepted 25–75%), and analysed them by repeated measures ANOVA.

#### Eye movement task

The eye movement task data were analysed according to a previously published approach (Muhammed *et al.*, 2016). Pupillometry analysis was performed using a one-way ANOVA to test for group differences in the variables of interest (CADASIL-apathy, CADASIL-no apathy and controls), and two-sided *t*-tests for subsequent comparisons of average pupil responsiveness between groups, with significance level set at *P*-values of < 0.05. Pearson correlations were used for parametric behavioural outcome comparisons. Time periods for averaging pupil responses were chosen based on past work (Muhammed *et al.*, 2016). Additionally, differences in pupillary reward sensitivity were analysed over time at each millisecond time point using multiple permutation testing (to account for multiple comparison bias).

#### Diffusion image preprocessing and analysis

All images were analysed in FSL (Smith *et al.*, 2004). Correction for susceptibility induced distortions, eddy currents and subject movement were performed using the FSL tools *topup* and *eddy* (Andersson *et al.*, 2003; Smith *et al.*, 2004; Andersson and Sotiropoulos, 2016) (Supplementary material). All images were visually inspected at each stage of processing.

White-matter voxelwise analysis of the fractional anisotropy imaging data was carried out using TBSS (Smith *et al.*, 2006) (Supplementary material). We used *randomise* (Winkler *et al.*, 2014) to carry out non-parametric voxelwise cross-subject statistics using 5000 permutations, and threshold-free cluster enhancement (TFCE) to correct for multiple comparisons across space. The *P*-value images for apathy < no apathy contrast in the CADASIL patients are displayed in the ‘Results’ section.

As a global metric of white matter integrity the mean fractional anisotropy for each participant was extracted from their skeletonized image, and group differences were tested for using a one-way ANOVA.

### Data availability

Anonymized data are available on request.

## Results

Demographic and mean values on questionnaire and baseline cognitive measures in the three groups are shown in Table 1 and Table 2, respectively. The percentage of CADASIL patients with apathy was 58%. CADASIL was associated with a reduced quality of life compared to controls, driven by the apathy group: *F*(2,37) = 8.7, *P* = 0.001; mean difference: CADISIL-apathy versus CADISIL-no apathy = 2.5, *P* = 0.01; mean difference: CADISIL-apathy versus healthy controls = 2.6, *P* = 0.001; mean difference: CADISIL-no apathy versus healthy controls = 0.1, *P* = 0.98. CADASIL was also associated with a higher level of depressive symptoms than controls, again driven by the apathy group: *F*(2,36) = 17.2, *P* < 0.001; mean difference: CADISIL-apathy versus CADISIL-no apathy = 4.7, *P* = 0.002; mean difference: CADISIL-apathy versus healthy controls = 5.9, *P* < 0.001; mean difference: CADISIL-no apathy versus healthy controls = 1.2, *P* = 0.53. Within the CADASIL group, apathy was not associated with significantly worse performance on baseline cognitive testing of executive functions, although they did trend towards a lower score on a global metric of cognition (ACE-III). There were no differences in motor signs between apathetic and non-apathetic patients.

**Table 2:** Baseline cognitive testing

**Measure**	**Control**	**CADASIL**	***P*-value**	**CADASIL No apathy**	**CADASIL Apathy**	***P*-value**
ACE	96.6 ± 2.4	92.3 ± 5.3	**0.003**	94.9 ± 2.4	90.3 ± 6.1	0.064
Digit Span		6.1 ± 0.8		6.1 ± 0.8	6 ± 0.8	0.74
Design Fluency	30.7 ± 6.6	25.4 ± 9.3	0.053	28.6 ± 5.4	23 ± 11.0	0.20
TMT − A^a^	N/A	30 ± 15.7	N/A	22.8 ± 8.0	35.8 ± 18.2	0.08
TMT − B^a^	N/A	86.7 ± 54.3	N/A	65 ± 27.1	104.1 ± 65.1	0.13
TMT − B-A^a^	N/A	56.7 ± 41.4	N/A	42.3 ± 25.2	68.3 ± 49	0.19

Values are mean ± SE. Values in bold represent *P* < 0.05. ACE = Addenbrooke’s Cognitive Examination III; TMT = Trail Making Task.

^a^Data for the Trail Making Task was missing for one participant.

### Apathetic CADASIL patients rejected more offers

**Figure 3:**
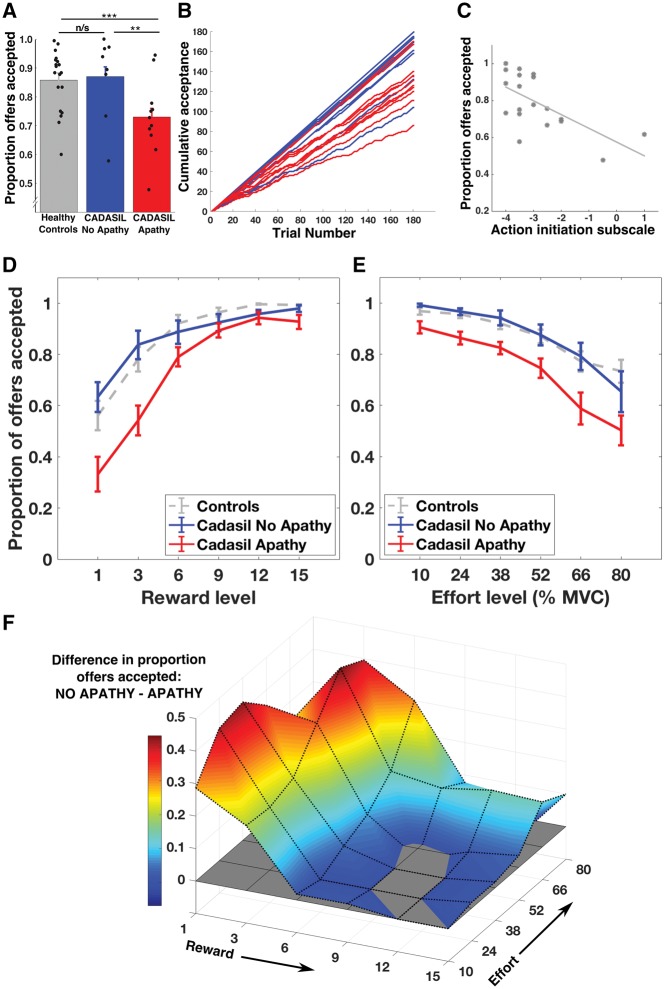
**Effort-based decision-making in CADASIL apathy.** (**A** and **B**) Apathetic patients accepted significantly fewer offers than non-apathetic patients or healthy controls. (**C**) The proportion of offers accepted was significantly correlated with the action initiation subscale of the LARS. (**D**) There was a significant apathy × reward interaction on offer acceptance. This plot shows the proportion of offers accepted as reward increases (collapsed across effort). Apathetic patients accepted fewer offers when rewarding outcomes were low, but performed the same as non-apathetic patients when rewards were high. (**E**) In contrast, there was no apathy × effort interaction. This plot shows offer acceptance as effort level increases (collapsed across reward). (**F**) The difference in offers accepted between no apathy and apathy groups occurred in a distinct region of decision space—where offers were for low rewards, irrespective of effort required. **A**, **D** and **E** show mean ± SE. **P < 0.05; ***P < 0.01; n/s = not significant.

First, we analysed the overall acceptance rates in the three groups using a one-way ANOVA. CADASIL patients with apathy accepted significantly fewer offers than patients without apathy or healthy controls, whilst acceptance rates did not differ between controls and non-apathetic patients [group effect: *F*(2,37) = 4.6, *P* = 0.017; mean difference: CADISIL-apathy versus CADISIL-no apathy = 0.14, *t*(17) = 2.5, *P* = 0.018; mean difference: CADISIL-apathy versus healthy controls = 0.13, *t*(17) = 2.77, *P* = 0.009; mean difference: CADISIL-no apathy versus healthy controls = 0.01, *t*(17) = 0.24, *P* = 0.81; Fig. 3A and B]. This global metric of offers accepted correlated significantly with the action initiation subscale of the LARS (*ρ* = 0.60, *n* = 19, *P* = 0.007; Fig. 3C).

### Reduced incentivization by rewards in apathetic patients

To investigate the factors driving reduced acceptance of offers in the apathetic group, we used a repeated measures ANOVA to analyse how the proportional acceptance rate in each cell of the sampled 6 × 6 decision space (Fig. 1C) varied with reward, effort and apathy. The difference in responding was not global across the sampled decision space, but rather varied with reward, but not effort. There was a significant two-way interaction between apathy and reward (Fig. 3D), but not between apathy and effort (Fig. 3E): Reward × Apathy, *F*(1.7,29.4) = 4.3, *P* = 0.028; Effort × Apathy, *F*(1.6,26.9) = 0.40, *P* = 0.63.

That is, apathetic patients rejected significantly more offers when reward was low, but showed no difference compared to non-apathetic patients at high reward levels, irrespective of what the effort costs were (Fig. 3F). There were also significant main effects of apathy [*F*(1,17) = 5.0, *P* = 0.038], reward [*F*(1.7,29.4) = 38.4, *P* < 0.001] and effort [*F*(1.6,26.9) = 21.7, *P* < 0.001], and a two-way interaction between reward and effort [*F*(5.9,100.2) = 3.6, *P* = 0.003]. There was no three-way interaction between apathy, reward and effort [*F*(5.9,100.2) = 1.3, *P* = 0.25].

**Figure 4:**
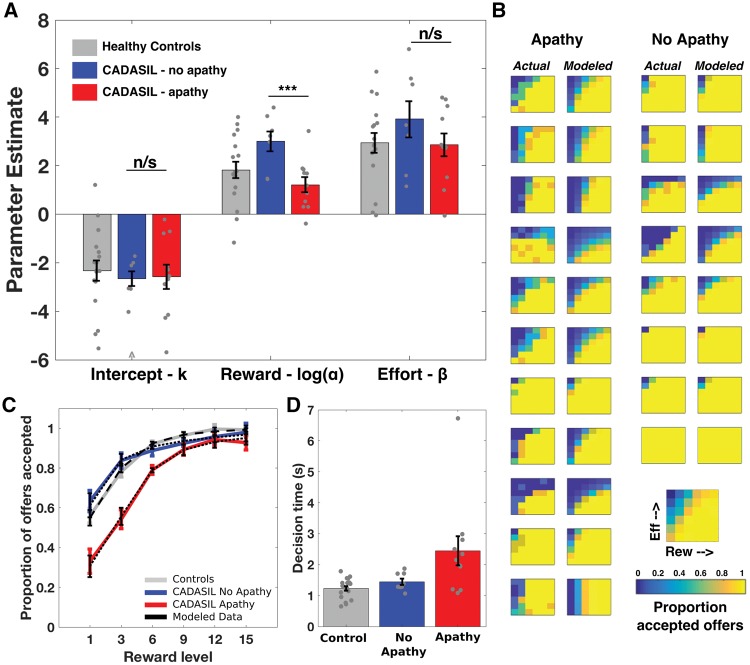
**Apathy in CADASIL and reward incentivization.** (**A**) The reward parameter (α) was significantly lower in apathetic compared to non-apathetic patients, whilst neither the effort parameter (β) nor intercept (κ) were significantly different. (**B**) Actual and modelled choice proportions for each apathetic (*left columns*) and non-apathetic (*right columns*) CADASIL patient. (**C**) Group level choice proportions with increasing reward, with model fits superimposed (black dotted lines). (**D**) Apathetic patients took longer to make accept/reject decisions than non-apathetic patients or healthy controls. Note that one patient (no apathy) and one control accepted all offers and had outlying (positive) intercepts. For clarity the parameter estimates for these participants are not plotted, however they were included in the analysis (inclusion or exclusion did not change any reported results). ^Outlying value not plotted for clarity (actual value = –18). ***P < 0.01; n/s = not significant.

Next we compared the parameter estimates for reward (α), effort (β) and intercept (κ) obtained from the computational model of choice fitted to each participant’s decisions (Fig. 4A). Modelled data closely approximated raw choice data, both for each CADASIL patient (Fig. 4B) and for group average choice proportions (Fig. 4C). The reward sensitivity parameter estimate—which quantified the degree that the rewarding outcome of the action increased the value of an offer—was significantly reduced in apathetic CADASIL patients compared to the non-apathetic patients {mean difference [log(*α*)] = 1.67, *t*(17) = 3.32, *P* = 0.004}. In contrast, there was no significant difference between apathy and no apathy patients for the effort sensitivity parameter estimate [the degree that effort reduced the value of an offer: mean difference (*β*) = 0.47, *t*(17) = 0.50, *P* = 0.62] or for the intercept [the baseline tendency to accept an offer: mean difference (*κ*) = 0.84, *t*(17) = 0.50, *P* = 0.63].

### Apathetic patients took longer to make effort-based decisions

The time each participant took to accept or reject an offer varied significantly by group, with both controls and non-apathetic CADASIL patients significantly faster than apathetic patients **[**main effect of group: *F*(2,33) = 5.8, *P* = 0.007; mean difference: CADISIL-apathy versus CADISIL-no apathy = 0.85 s, *t*(17) = 2.04, *P* = 0.05; mean difference: CADISIL-apathy versus healthy controls = 1.1 s, *t*(28) = 3.39, *P* = 0.002; mean difference: CADISIL-no apathy versus healthy controls = 0.27s, *t*(25) = 0.70, *P* = 0.49; Fig. 4D]. In contrast to this effect, apathetic patients were not significantly slower than non-apathetic patients on a neuropsychological measure of processing speed (Trail-Making Task B – A, *P* = 0.2, Table 1), nor did they show differences in reaction time when performing the eye-tracking task (*P* = 0.69, Supplementary Fig. 3C). Both apathetic and non-apathetic CADASIL patients showed the expected modulation of decision time by choice difficulty, taking longer to make hard compared to easy choices: *F*(1,29) = 44.76, *P* < 0.001; mean difference (hard - easy): CADISIL-apathy = 0.41 s, *t*(9) = 4.17, *P* = 0.002; mean difference: CADISIL-no apathy = 0.74s, *t*(5) = 3.93, *P* = 0.01. This difficulty effect was not significant in the control group [mean difference: healthy controls = 0.1s, *t*(15) = 1.43, *P* = 0.17], leading to an interaction between group and difficulty [*F*(2,29) = 9.1, *P* = 0.001; Supplementary Fig. 2A].

Participants in both CADASIL groups and the control group parametrically modulated their force output to task requirements, with no force × group interaction [main effect of force level: *F*(1.8,60.2) = 517.5, *P* < 0.001; force × group interaction *F*(3.5,60.2) = 1.4, *P* = 0.26, Fig. 1E]. Participants on average achieved required force levels >95% of the time (mean success rate for healthy controls, CADISIL-no apathy and CADISIL-apathy = 98.6%, 96.2% and 96.7%, respectively [CADISIL-apathy versus CADISIL-no apathy: mean difference = 0.5%, *t*(17) = 0.3, *P* = 0.75, Supplementary Fig. 2B]. Participants’ choices did not systematically change across the course of the experiment, with no effect of block number on acceptance rate in control [*F*(4,179) = 0.41, *P* = 0.8], non-apathetic [*F*(4,179) = 0.81, *P* = 0.52] or apathetic participants [*F*(4,179) = 0.15, *P* = 0.96, Supplementary Fig. 2C–E]. Furthermore, although apathetic patients reported higher levels of depressive symptoms (Table 1), there was no main effect of depression on proportion of offers accepted [*F*(1,16) = 2.2, *P* = 0.16], nor any interactions between depression and either reward or effort [Reward × Depression: *F*(1.6,25.4) = 0.47, *P* = 0.59; Effort × Depression: *F*(1.5,24.8) = 0.65, *P* = 0.49; Supplementary material].

### Apathetic CADASIL patients showed reduced autonomic responses to reward

**Figure 5:**
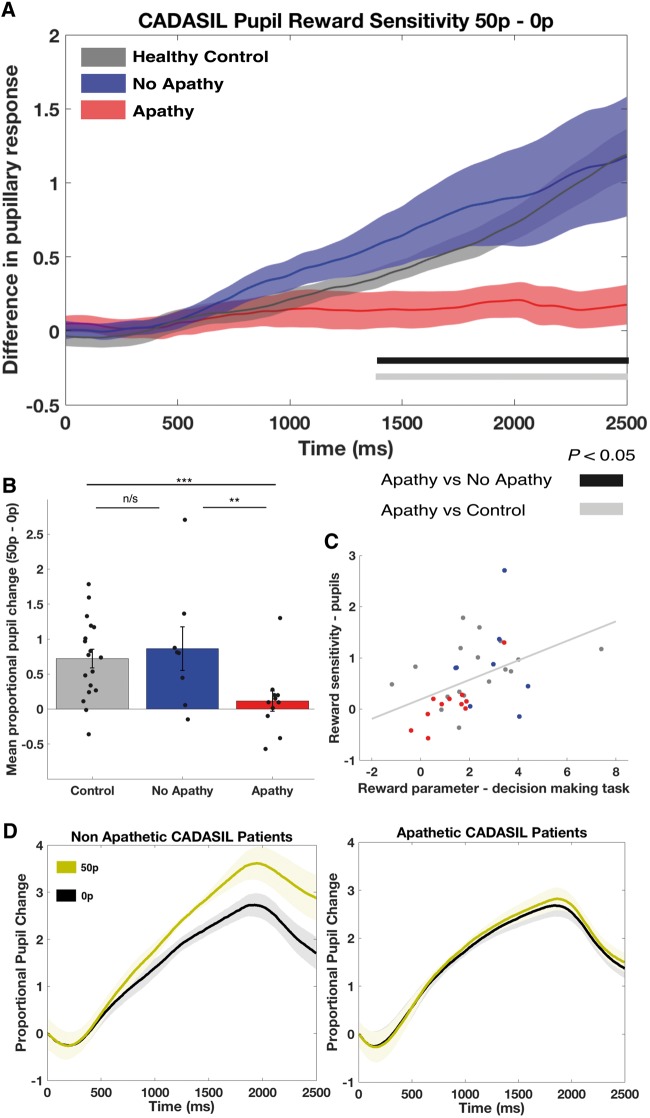
**Autonomic responses to reward in CADASIL apathy.** (**A**) Apathetic CADASIL patients had a significantly diminished pupillary response to increasing incentives compared to both non-apathetic patients and healthy controls. Reward sensitivity (indexed as the difference in pupil dilatation between the high and no reward conditions) was assessed at each millisecond time point using multiple permutation testing (to correct for multiple comparisons). Apathetic patients had significantly blunted pupil responses compared to non-apathetic patients and healthy controls from ~1380 ms to the end of each trial. (**B**) Mean reward sensitivity (time period 1400–2400 ms) was significantly lower in the apathetic group compared to non-apathetic patients and controls. (**C**) Physiological reward sensitivity, as indexed by pupillary response to reward, was significantly correlated with the reward parameter estimate (α) from the effort-based decision-making task; red dots = CADASIL apathy; blue dots = CADASIL no apathy; grey dots = controls. (**D**) Actual (proportional) changes in pupil dilatation for high (gold) and no (black) reward conditions, for non-apathetic and apathetic groups. Both groups demonstrate significant pupillary dilatation to auditory cue, however in the apathy group this response is not modulated by reward level. Mean ± SE shown in **A** and **B**. **P < 0.05; ***P < 0.01; n/s = not significant.

Apathy in CADASIL was associated with blunted pupillary responses to reward, as measured by the proportional change in pupil size between high reward (50p) and low reward (0p) conditions of the eye movement task (Fig. 5A and B). There was a significant main effect of group on this metric: *F*(2,34) = 4.3, *P* = 0.021. This was driven by a reduced change in pupil size as reward increased in the apathy group, compared to both the no apathy group [mean difference in proportional change = 0.75 ± 0.29, *t*(17) = 2.39, *P* = 0.029] and the healthy matched controls [mean difference = 0.61 ± 0.24, *t*(27) = 2.94, *P* = 0.007; Fig. 5B]. There was no difference between non-apathetic CADASIL patients and controls [mean difference = 0.14 ± 0.27, *t*(24) = 0.49, *P* = 0.63]. Importantly, there was no significant difference in baseline pupil size between groups [*F*(2,34) = 0.68, *P* = 0.51], meaning the reduced response associated with apathy could not simply be explained by these patients having greater dilatation at the start of the experiment. Furthermore, there was no general deficit in pupil motility in the apathetic group (Fig. 5D). There was also no significant effect of depression on the proportional change in pupil size [mean difference (low depression - high depression) = 0.44 ± 0.37, *t*(16) = 1.2, *P* = 0.25].

The degree of pupillary dilation to rewards was significantly correlated with the behavioural metric of reward sensitivity derived from the effort-based decision-making task (R = 0.45, *n* = 36, *P* = 0.006; Fig. 5C). Therefore, a reduction in reward incentivization on the decision-making task was associated with reduced physiological responses to reward on an independent eye movement task, and both these metrics were significantly diminished in the apathy group.

### Apathy not associated with changes in saccadic velocity

There was no significant difference in peak saccadic velocity between apathetic and non-apathetic CADASIL patients or healthy controls [group difference (repeated measures ANOVA): *F*(2,34) = 0.24, *P* = 0.79; mean (no apathy) = 545 ± 25°/s; mean (apathy) = 523 ± 21°/s, *P* = 0.58]. There was a significant main effect of reward on velocity, with participants reaching a higher peak saccadic velocity as reward level increased [mean 0p = 525°/s; mean 10p = 531°/s; mean 50p = 534°/s, *F*(2,68) = 6.8, *P* = 0.002; Supplementary Fig. 3A]. There was no Reward × Group interaction, indicating this effect was present across all groups: *F*(4,68) = 1.43, *P* = 0.24. There was also no correlation between reward sensitivity indexed by change in saccadic velocity (50p – 0p) and the reward parameter estimate from the decision-making task (R = 0.074, *n* = 36, *P* = 0.678; Supplementary Fig. 3B).

### Reduced white matter integrity in CADASIL apathy: tract-based spatial statistics

**Figure 6:**
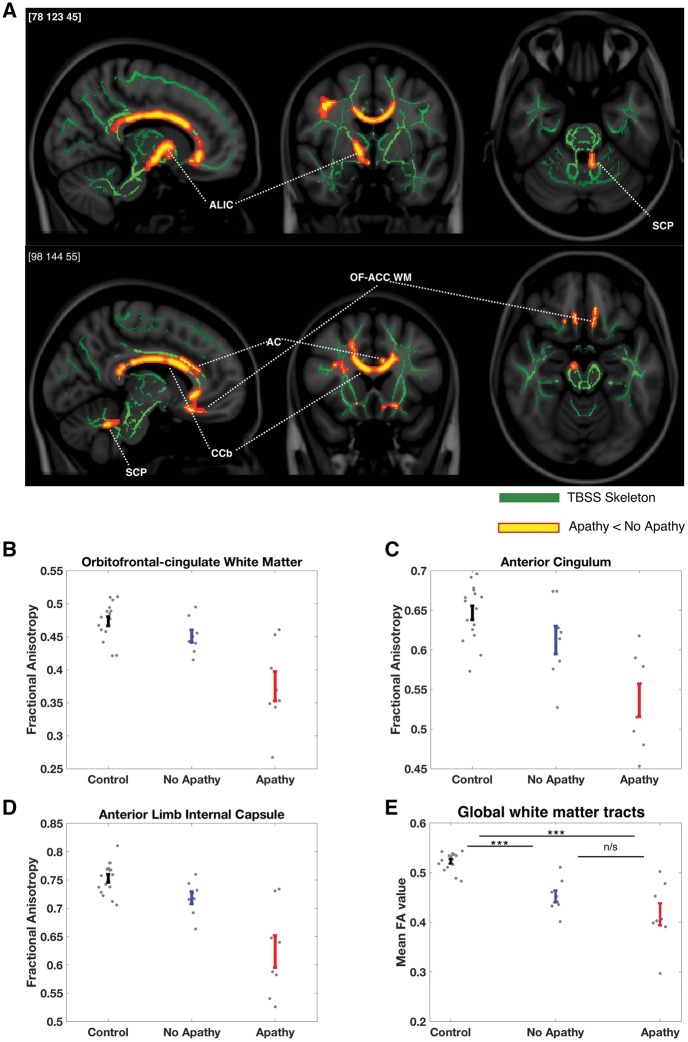
**Changes in white matter tract integrity associated with apathy in CADASIL.** (**A**) Apathetic CADASIL patients (*n* = 8) had significantly reduced white matter fractional anisotropy compared to non-apathetic CADASIL patients (*n* = 8) within the right anterior cingulum (AC), bilateral orbitofrontal-anterior cingulate white matter (OFC-ACC WM), right anterior limb of the internal capsule (ALIC), body of the corpus callosum (CCb) and left superior cerebellar peduncle (SCP): *P* < 0.05, TFCE-corrected in red-yellow, against the study-specific white matter tract skeleton in green. (**B**–**D**) The associated error bar plots (mean ± SE) show the average fractional anisotropy values from significant regions (from the apathy versus no apathy contrast) for control (*n* = 16) and CADASIL patients. Fractional anisotropy in the apathy group appears to be reduced compared with both non-apathetic patients and controls at each location, whereas control and non-apathetic patient values overlapped considerably. (**E**) In contrast, global mean fractional anisotropy values were reduced in both apathetic and non-apathetic CADASIL patients compared to controls, but did not significantly differ from each other.***P < 0.01; n/s = not significant.

Three apathetic CADASIL patients were excluded from the imaging analysis [because of claustrophobia (one), excessive movement (one) and an acute abnormality on the scan (one)]. This left 16 CADASIL patients (eight apathetic, eight non-apathetic), and their 16 matched controls. Apathy was associated with significantly reduced fractional anisotropy in the left anterior cingulum, bilateral orbitofrontal-anterior cingulate white matter tracts, right anterior internal capsule, the body of the corpus callosum and the left superior cerebellar peduncle (Fig. 6A). There was also a significant reduction within a region of the right corona radiata. There were no regions where fractional anisotropy was significantly higher in apathetic compared to non-apathetic CADASIL patients.

We used these white matter regions showing significant differences between apathetic and non-apathetic patients to define masks at each location. Mean fractional anisotropy values extracted from these masks for the healthy controls and both patient groups suggest that disruptions in these white matter tracts were specific to the apathetic status of the CADASIL patients (Fig. 6B–D and Supplementary Fig. 4). Furthermore, apathetic and non-apathetic CADASIL patients did not differ in mean fractional anisotropy extracted across the entire TBSS skeleton [mean difference (fractional anisotropy) = 0.036, *P* = 0.15]. Rather, there was a general effect of disease with fractional anisotropy in both groups significantly lower than the controls [mean difference (no apathy versus controls) = 0.071, *P* < 0.001; mean difference (apathy versus controls) = 0.11, *P* < 0.001; Fig. 6E]. Finally, we tested whether fractional anisotropy values within the apathy-associated regions were associated with behavioural reward sensitivity. Fractional anisotropy values within orbitofrontal-anterior cingulate (OFC-ACC) white matter and the anterior limb of the internal capsule significantly predicted behavioural reward sensitivity, after first controlling for the effect of apathy. That is, patients with reduced white matter integrity within these regions also had lower sensitivity to rewarding outcomes on the decision-making task (OFC-ACC: ΔR^2^ = 0.2, F = 5.3, *P* = 0.04; ALIC: ΔR^2^ = 0.3, F = 5.8, *P* = 0.03; Supplementary material).

## Discussion

This study provides evidence that disrupted effort-based decision-making underlies apathy in patients with CADASIL, with reduced reward sensitivity being a key marker that distinguished apathetic from motivated patients. Overall, apathetic individuals rejected more offers of reward for effort, but this change was not global across the sampled decision space. Rather it manifested only in a certain region of the reward-effort decision space: where rewarding outcomes were low (Fig. 3). Computational modelling of these decisions confirmed this change in responding was driven by reduced reward incentivization in the apathy group (Fig. 4), a finding that was mirrored by the blunted autonomic responses to rewards in these same patients as indexed by pupillary response to potential rewards (Fig. 5). Furthermore, white matter integrity was reduced within the apathy group specifically within tracts connecting neural regions crucial for reward processing and effort-based decision-making (Fig. 6).

Together these results demonstrate, through converging behavioural, physiological and anatomical methods, evidence for a specific cognitive mechanism underlying the reduced goal-directed behaviour that defines apathy in CADASIL. The concordance of the imaging findings with a previous study of apathy in sporadic SVD (Hollocks *et al.*, 2015) suggest that these results may also be translatable to SVD more generally. Furthermore, the similar behavioural and physiological findings previously demonstrated in Parkinson’s disease (Muhammed *et al.*, 2016; Le Heron *et al.*, 2018) point to disrupted effort-based decision-making as a cognitive mechanism that may underlie apathy, across neurodegenerative diseases.

The change in effort-based decision-making associated with apathy manifested in the behavioural task as reduced engagement with a subset of offers—those in which the rewarding outcomes were low. Put another way, apathetic patients were prepared to exert as much effort as non-apathetic patients and healthy controls, even at high effort requirements, if the reward was high. This suggests that when apathetic patients fail to engage with a potential activity, it is not because the costs associated with acting are too high, but rather the outcome value of the action is not sufficient to drive behaviour towards it. Importantly, this reward insensitivity was also evident in the physiological responses of apathetic patients to reward. Their autonomic (pupil) responses to increasing reward levels were blunted in comparison to both non-apathetic patients and healthy controls. Furthermore these two markers of reward sensitivity (behavioural and physiological) were themselves correlated across all participants, consistent with them both measuring the same reward metric.

Importantly, non-apathetic patients and healthy controls did not differ in their behavioural or autonomic responses. Therefore, the observed changes were not simply a general feature of having CADASIL, and nor could they be explained by other confounders such as age or gender, which were matched between groups, or depression, which was not associated with significantly altered responses. Rather, reduced incentivization by reward was a specific feature of the apathetic state. Evidence for such reward insensitivity in apathy has previously been demonstrated following stroke (Rochat *et al.*, 2013) and in Parkinson’s disease (Lawrence *et al.*, 2011; Martinez-Horta *et al.*, 2014; Muhammed *et al.*, 2016; Le Heron *et al.*, 2018). It suggests that the failure to activate goal-directed behaviour in apathy may relate to altered processing of reward information about potential actions (Levy and Glimcher, 2012; Rushworth *et al.*, 2012), or in the subsequent integration of reward with effort costs to drive behaviour (Holroyd and Yeung, 2012).

Not all aspects of the reward-related response were significantly altered in patients with apathy. Specifically, peak saccadic velocities to the rewarding target were no different compared to either non-apathetic patients or healthy controls, an observation that has also been reported in Parkinson’s disease (Muhammed *et al.*, 2016). Similarly, in the behavioural decision-making task their motor responses to the rewarding target were also normal. Therefore, although apathetic patients were less sensitive to reward value (whether indexed by choice or pupillary dilatation), once they had accepted an offer (behavioural task) or were cued to saccade (eye tracking task), their motor responses were unimpaired. One frequently emphasized feature of apathy is that it is a failure of self-generated goal-directed behaviour (Levy and Dubois, 2006; Sockeel *et al.*, 2006; Robert *et al.*, 2009). In both the paradigms used in the current study, motor responses were externally cued (by the appearance of a force level on a cartoon tree or a peripheral visual target, respectively), which may have effectively bypassed the disrupted mechanisms underlying apathy. This demonstrates that not all components of effortful behaviour for reward are disrupted in apathy and points to a potentially important, simple therapeutic strategy—providing external cues or prompts for action within an apathetic patient’s environment.

Prominent theories of behaviour have identified separate systems that can lead to actions. Whilst goal-directed systems explicitly represent information about action outcomes (rewards) and costs in determining whether to engage, habitual systems rely on simpler, previously learnt stimulus-response associations (Balleine and O’Doherty, 2010). Indeed, the technique of behavioural activation, which is thought to work via the same processes underlying habitual systems (instrumental conditioning), has been shown to be effective in treating depression (Ekers *et al.*, 2014). Because of the relative ease of instituting such interventions, in our view, they would certainly be worth exploring further as potential non-pharmacological treatments for apathy. Such a strategy obviates the need for an effort-based decision to be made, activating behaviour independent of goal-directed systems, and could theoretically be tailored to an individual’s psychosocial backdrop and previous interests, for example directing behaviour towards hobbies that they have previously engaged with.

Another potential therapeutic strategy that might be important to explore is the possibility of increasing reward sensitivity. The findings in this study demonstrate that apathetic patients are not incentivized by low reward outcomes—the types of outcome associated with many of the everyday activities these patients fail to engage with. Modulating the reward system to amplify the incentivizing effects of reward could be achieved pharmacologically, with a number of neuromodulatory systems providing potential targets. This has been shown to occur with dopaminergic therapy in Parkinson’s disease (Le Bouc *et al.*, 2016; Muhammed *et al.*, 2016; Le Heron *et al.*, 2018) and following basal ganglia stroke (Adam *et al.*, 2013), whilst manipulation of serotonergic (Callegari *et al.*, 2016) and cholinergic (Devos *et al.*, 2014) systems, which are also closely linked to effort-based decision-making (Nunes *et al.*, 2013), has also been shown to improve apathy. Whether such manipulations would have any impact on apathy in CADASIL or sporadic SVD remains to be established.

Apathetic patients with CADASIL were slower to make decisions about whether to accept or reject offers. Although this could be caused by a general slowing in processing speed associated with damage to frontal white matter connections (O’Sullivan *et al.*, 2005), there were no significant differences in performance either on the paper-based trail making test, or reaction time for saccade initiation in the eye tracking task. This suggests that, in fact, the increased decision latency is a feature of changes in the decision process itself, consistent with the broader hypothesis of disrupted effort-based decision-making underlying apathy.

Anatomically, apathy was associated with reduced white matter integrity within specific regions: anterior cingulum, orbitofrontal-anterior cingulate white matter, anterior limb of the internal capsule and the body of the corpus callosum. Furthermore, white matter integrity in two of these regions (orbitofrontal-anterior cingulate white matter and anterior limb of internal capsule) was directly associated with behavioural reward sensitivity, after controlling for the presence of apathy. In contrast, apathy was not associated with overall burden of white matter disease (assessed by each participant’s mean global skeletonized fractional anisotropy value). Instead this metric was altered as a function of disease itself. These findings link the behavioural and physiological evidence of disrupted effort-based decision-making in CADASIL apathy to disruption of tracts that connect brain areas strongly implicated in this cognitive process (Prevost *et al.*, 2010; Neubert *et al.*, 2015; Klein-Flugge *et al.*, 2016; Hauser *et al.*, 2017).

These anatomical findings are consistent with studies of apathy in different conditions. The anterior cingulum is closely associated with the overlying anterior cingulate cortex, and reduced fractional anisotropy within this tract has been linked to apathy in sporadic SVD (Hollocks *et al.*, 2015) and Alzheimer’s disease (Kim *et al.*, 2011), as well as reduced motivation in healthy people (Bonnelle *et al.*, 2016). The identified ventral white matter region associated with OFC-ACC lies medial to the uncinate fasciculus and may represent the amygdalofugal pathway, a tract that links amygdala and regions including ventral striatum and medial frontal cortex (Miller *et al.*, 2010; Mori *et al.*, 2017). The anterior limb of the internal capsule contains fibres projecting between anterior and mediodorsal thalamus and prefrontal and anterior cingulate cortex and has recently been associated with apathy in frontotemporal lobar degeneration (Lansdall *et al.*, 2018). Strokes within these regions of the thalamus are also an important subcortical cause of apathy (Carrera and Bogousslavsky, 2006). Reduced fractional anisotropy in the body of the corpus callosum has been associated with apathy in progressive supranuclear palsy (Agosta *et al.*, 2014) and other frontotemporal dementias (Lansdall *et al.*, 2018). Furthermore, the medial cortical and subcortical regions connected by these tracts have been consistently associated with apathy across multiple disorders and imaging modalities (Lanctôt *et al.*, 2007; Schroeter *et al.*, 2011; Huang *et al.*, 2013; Le Heron *et al.*, 2017). It is within these regions that the core processes of effort-based decision-making to drive motivated behaviour are thought to be instantiated (Migneco *et al.*, 2001; Croxson *et al.*, 2009; Kennerley *et al.*, 2009; Schroeter *et al.*, 2011; Levy and Glimcher, 2012; Rushworth *et al.*, 2012; Le Heron *et al.*, 2017).

Although speculative, these apathy-associated white matter changes may provide a clue as to why reward modulation of pupil response is reduced in apathetic CADASIL patients. Pupil dilatation is influenced by noradrenergic projections arising with the locus coeruleus (Hou *et al.*, 2005). However, the fact apathetic patients do not show a general deficit in pupil motility suggests the changes observed in this group may be more upstream. The locus coeruleus receives dopaminergic projections from the ventral tegmental area (VTA) (Samuels *et al.*, 2006). In turn, the VTA has widespread afferent projections from prefrontal and cingulate cortex, subcortical and brainstem structures, which influence the firing rate of VTA neurons and subsequent dopamine release (Haber and Knutson, 2010). Additionally, it has been demonstrated previously that ACC and OFC lesions impair effort and reward-related autonomic arousal in both primates and humans (Critchley *et al.*, 2003; Reekie *et al.*, 2008; Manohar and Husain, 2016). Any compromise of projections from these regions to VTA, such as secondary to the reduced white matter integrity present in the apathy group, could theoretically alter regulation of locus coeruleus neurons by the VTA, leading to the observed loss of modulation by reward in the pupil responses of apathetic CADASIL patients.

In interpreting the findings from this study it is important to consider strengths and potential limitations. The lack of significant differences on measures of executive function as well as the specific rather than general changes in decision-making associated with apathy suggest the observations in the decision-making paradigm are unlikely to be explained by a confounding cognitive problem. Further, the apathetic group modulated their force production to force requirements, and showed the expected relationship between decision time and choice difficulty. The study protocol controlled for other potential confounders such as fatigue and temporal discounting. Baseline pupil size did not differ by group in the eye-tracking task, and the normal saccadic responses in the apathetic group is consistent with them attending to the task in the same manner as other participants. The sample size of 19 patients with CADASIL (and 16 for the imaging component) is a potential limitation and the findings in the study should be weighted against this. Although we note that it is difficult to perform extensive behavioural and imaging studies in this rare patient group, the results would merit replication in a different sample. It will be important in particular to replicate the tractography findings. However, CADASIL remains a rare condition (Chabriat *et al.*, 2009) and there is no reason to consider the sampled population to be unrepresentative. Additionally, by sampling a smaller group we were able to perform a more in-depth, mechanistically focussed experiment, in a population that provides a model of pure vascular cognitive impairment (Charlton *et al.*, 2006). The concordance of results across modalities strengthens the conclusions we can draw, as do the strong parallels we found between the behavioural and physiological responses of apathetic CADASIL patients and Parkinson’s disease patients performing the same tasks (Muhammed *et al.*, 2016; Le Heron *et al.*, 2018).

This study provides behavioural, physiological and anatomical evidence that disrupted effort-based decision-making underlies apathy in patients with CADASIL. Specifically, apathetic responses were characterized by reduced incentivization by the rewarding outcomes of actions. These changes manifested in apathetic patients rejecting lower reward offers in the decision-making task—offers that likely correspond to the rewarding outcomes of many everyday activities. This suggests that the reduced goal-directed behaviour that characterizes apathy occurs not because of the costs of making actions, but because patients do not derive sufficient drive from the outcomes of these actions. The concordance of the results with studies in other populations fits with a transdiagnostic model of apathy, in which the syndrome develops due to disruption of specific cognitive mechanisms (here reward incentivization and effort-based decision-making), irrespective of the underlying disease state. The reduced quality of life associated with apathy in this and other studies underlines the importance of developing treatments for it—treatments that this work suggests should be targeted at increasing the incentivising value of rewards.

## Supplementary Material

Supplementary Data

## Abbreviations

ACC: anterior cingulate cortex
CADASIL: cerebral autosomal dominant arteriopathy with subcortical infarcts and leukoencephalopathy
OFC: orbitofrontal cortex
SVD: cerebral small vessel disease
