# Supplementary material for: Dysfunctional effort-based decision-making underlies apathy in genetic cerebral small vessel disease
Source: Brain. 2018 Oct 20;141(11):3193–210. doi: 10.1093/brain/awy257 (PMC6202575; doi:10.1093/brain/awy257)
Supplement: Supplementary Data [file awy257_suppl_data.pdf]

SUPPLEMENTARY MATERIAL | Le Heron *et al*

EFFORT-BASED DECISION MAKING TASK

Participants were administered an effort-based decision making task, which has been previously used to investigate apathy in patients with Parkinson's disease (Le Heron 2018). They were seated in front of a desktop computer running Psychtoolbox (psychotoolbox.org) implemented within Matlab (MathWorks, USA). They registered their responses using one of two handheld dynamometers (SS25LA, BIOPAC Systems, USA), which were calibrated to each individual at the beginning of the session. Each participant's maximal voluntary contraction (MVC) was calculated separately for each hand, defined as the maximum force exerted over three maximal contractions. All subsequent responses were thus normalised to each individual.

On a trial-by-trial basis, participants were given sequential offers of reward in return for exerting effort (**Figure 1a**). Each offer was presented on the screen as a cartoon apple tree. Reward on offer for the current trial was indicated by the number of apples on the tree (1, 3, 6, 9, 12 or 15) and was also numerically displayed underneath the tree. Each apple was worth 1p. Effort required to obtain the reward was indicated by the height of the yellow bar on the tree trunk, with the six possible levels corresponding to 10, 24, 38, 52, 66 and 80% of a participant's MVC (**Figure 1b**). The six reward and effort levels were systematically combined and the resultant 36 conditions were sampled evenly in a pseudo-randomised order across five blocks, for a total of 180 trials (**Figure 1c**). This meant all participants received the same offers, presented in the same order.

Participants were instructed to weigh up the effort costs against the reward on offer for each trial, and decide *"if it is worth it – is it worth squeezing that hard for that number of apples?"* If they accepted an offer (by exerting a small squeeze on the left hand-grip) then they had to squeeze to the required force and hold above this level for at least one second, within a five second response window. During the squeeze, online force feedback was shown as a red bar that indicated current force relative to the target line. If successful, they were 'rewarded' with the apples on offer, seeing the message *"xxx apples gathered"* as well as a cumulative tally of apples gathered on the current block *("total apples in basket: xxx")*. If unsuccessful the message displayed was *"0 apples gathered"*. Conversely, if participants rejected an offer (by

exerting a small squeeze on the right-hand grip) they waited an equivalent time (to control for temporal discounting effects) before moving onto the next offer. Therefore, on each trial participants decided whether the value of an offer was worth engaging with, compared to doing nothing for the equivalent time (**Figure 1a**). Before starting the experiment, participants practised each force level with each hand to familiarise themselves with the effort required, and completed a practice block in which they made decisions on the full range of options in the experiment.

We included a number of features to reduce potential effects of fatigue on choice. Both hands were used for the experiment. The required response side was randomised, and signalled on each trial *after* an offer was accepted, by the apple tree moving from the centre position to the left (left hand) or right (right hand) side of the screen, selected pseudo-randomly on each trial. Additionally, 25% of accepted offers did not require a squeeze (although subjects were instructed to make their responses assuming they would have to exert effort if accepted); in these cases (which were pseudo-randomly distributed throughout the experiment) subjects waited the equivalent time as if they had rejected the offer. Finally, subjects were allowed to rest between each block of 36 trials until they felt ready to continue (generally 30 – 120 seconds).

EYE TRACKING PARADIGM

The eye tracking task used in this study has been described in detail in a previous publication, when it was used in a cohort of patients with Parkinson’s disease (Muhammed et al., 2016). The task involved participants performing saccadic eye movements from a central fixation point to a peripheral target to earn monetary rewards. Three levels of reward were used – 0p, 10p and 50p. These values were the maximum amount that could be earnt on the current trial, and were conveyed to participants via an audio cue once they had maintained central fixation for 500ms. Then, following a period of 1400, 1500 or 1600ms, the fixation cue disappeared and concurrently a peripheral target cue appeared, randomised between left and right, at an angle of 11°. This was the participant’s cue to saccade as quickly as possible to the new target. Participants were aware that the proportion of the maximal earnings they were paid depended on how quickly they reached the target. Although the absolute reward earned varied based on this time, the amount was dynamically adjusted for each participant via an adaptive exponential fall-off based on their average time on the preceding 20 trials. Therefore difficulty level remained consistent across the experiment, accounting for potential

confounding factors such as fatigue and baseline reaction time, whilst also ensuring equal rewards were earned by all participants. After reaching the cue, visual feedback about the amount earned on that trial was displayed within the target, as well as an audible bell sound if they earned more than 10p, and a cash register sound if they earned more than 30p. No sound was played for less than 10p earned. Participants performed five blocks of 54 trials each, with the three reward levels interleaved through each block. They were given a 5-min break between the first three and last two blocks. In total each participant completed 270 trials, with 90 trials for each of the three reward levels.

An infrared eye tracker (Eyelink 1000, SR Research) was used to monitor pupil diameter and eye position. Disc luminance was matched across all trials so as not to affect pupil dilation. Eye tracking was performed in a dimly lit room 60 cm in front of a 21” CRT (1024 × 768 pixels; 100 Hz refresh). Stimuli were presented on a Windows computer running Matlab (The MathWorks) and Psychophysics Toolbox. The frame-mounted infrared tracker monitored left eye position and sampled at 1 kHz. Eye movements were measured online by the Eyelink computer and transferred directly to the presentation computer to provide immediate feedback. Nine-point calibration was performed. Pupil dilation was calculated as a proportional change from average baseline pupil size measured in Eyelink units. Recordings were time locked to the reward cue onset and normalized using a 200 ms baseline subtraction for each trial. Pupil traces lost due to blinks were interpolated. A moving average smoothing window of 100 ms was applied to the final recordings. Eye movement analysis was carried out using custom-made Matlab code. An eye movement was classified as a saccade if it was >2°, and the accepted landing area to register completion of the saccade was 5° in radius from target centre. The first landing point within the target area was used to classify saccade completion. Reaction time was calculated from target onset to the time when a saccade was registered as complete, and peak saccadic velocity computed as maximum velocity recorded during the saccade.

COMPUTATIONAL MODEL OF CHOICE

Candidate models were selected based on prior decision making literature (Chong et al., 2017; Prevost et al., 2010) and intuition about how effort would affect the value of a reward. Four general categories of model (Linear, Quadratic, Exponential and Hyperbolic) were compared using standard methods (Bayesian information criterion (BIC) and visual inspection of individual and group average fits to the raw data. Each model ascribed a particular subjective value to each reward/effort combination, which varied depending on the parameters associated with reward and effort (fitted for each participant separately). A softmax function transformed this value to a value between 0 and 1 – the estimated probability of acceptance. Parameter estimates for reward, effort and an intercept were calculated using *fminsearch* (Matlab, Mathworks, USA) such that the difference between a person's actual and modelled choice for each trial was minimised.

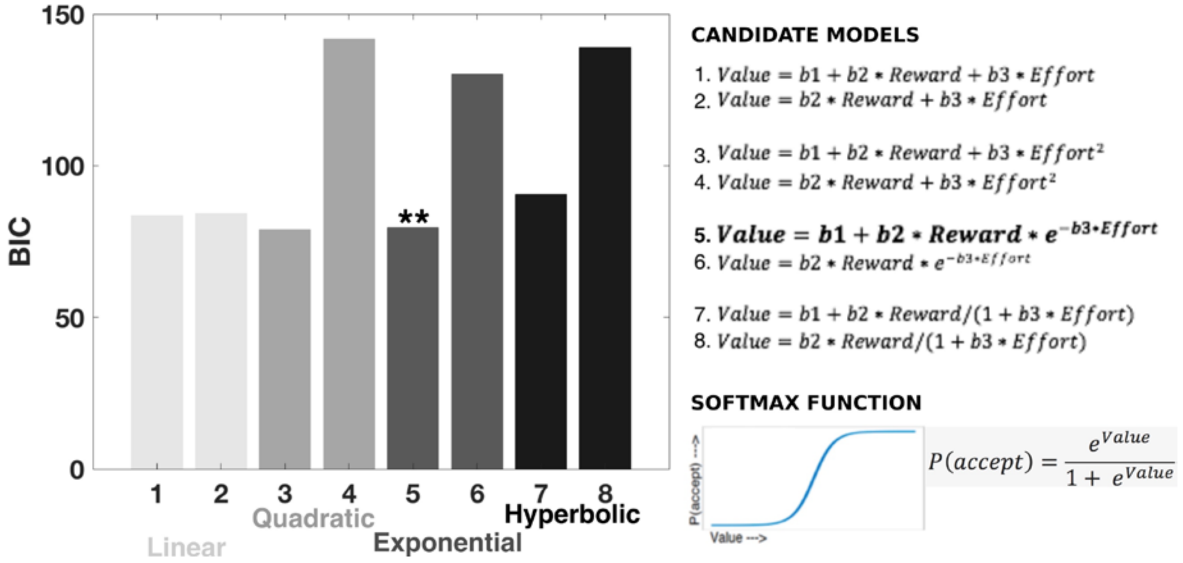

Supplementary figure 1. Model comparison for analysis of decision making choice data. Candidate models were compared using median BIC value and visual inspection of model fits. The softmax function computed the probability of accepting an offer given its value. Model 5 was the winning model.

DIFFUSION IMAGE PRE-PROCESSING

Correction for susceptibility induced distortions, eddy currents and subject movement:

This was performed using the FSL tools *topup* and *eddy*. Diffusion data was collected with reversed phase-encode blips, resulting in pairs of images with distortions going in opposite directions. From these pairs the susceptibility-induced off-resonance field was estimated using a method similar to that described in (Andersson et al., 2003) as implemented in FSL (Smith et al., 2004) and the two images were combined into a single corrected one. The output from *topup* was then passed to *eddy* (Andersson and Sotiropoulos, 2016) to correct for distortions associated with eddy currents and for subject movement. All images were visually inspected at each stage of processing.

TRACT-BASED SPATIAL STATISTICS (TBSS):

Voxelwise statistical analysis of the FA data was carried out using TBSS (Smith et al., 2006). First, FA images were created by fitting a tensor model to the raw diffusion data using *DTIFIT* within FMRIB's diffusion toolbox (FDT). FA data from all subjects was then aligned into a common space (FMRIB58_FA) using the nonlinear registration *FNIRT* (Andersson et al., 2007). The mean (across all participants) FA image was then created and thinned to create a mean FA skeleton, representing the centre of all tracts common to the group. Each subject's aligned FA data was then projected onto this skeleton, and thresholded using a standard value of 0.2. Non-parametric voxelwise cross-subject statistics were then performed with *randomise* (Winkler et al., 2014) using threshold free cluster enhancement (TFCE) and 5000 permutations.

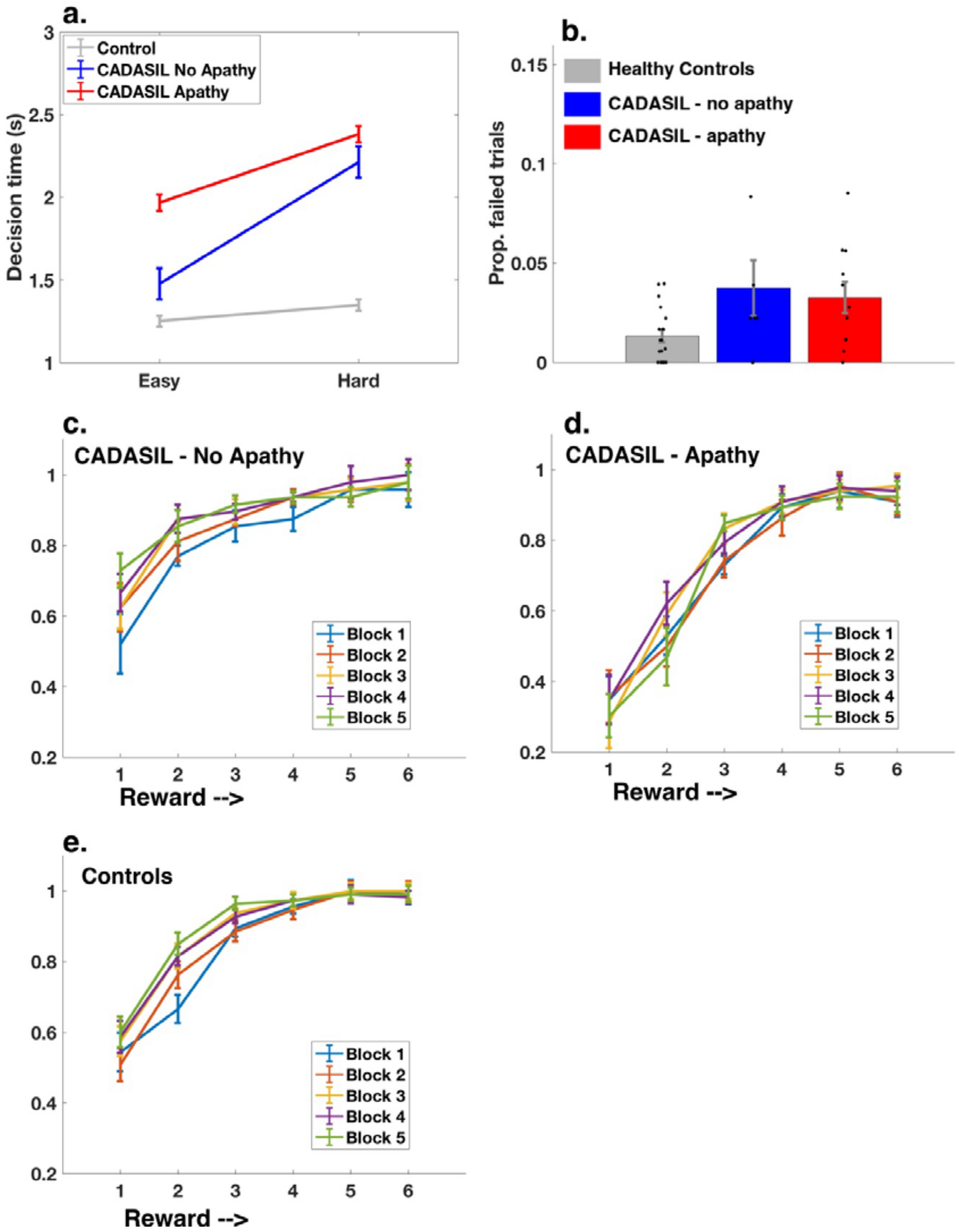

Supplementary figure 2.

Both apathetic and non-apathetic patients' decision times varied with decision difficulty, defined as the proportion of offers accepted (easy = <0.25 or > 0.75; hard = 0.25-0.75). Both apathetic and non-apathetic CADASIL patients took longer to make hard compared to easy choices. This effect was not significant in the control group. **(a)**. All groups achieved the required force > 95% of the time on accepted trials, with no difference between apathetic and non-apathetic CADASIL patients (**b**). The proportion of offers accepted at each reward level

did not vary systematically across the experiment in non-apathetic CADASIL patients (**c**), apathetic CADASIL patients (**d**) or controls (**e**).

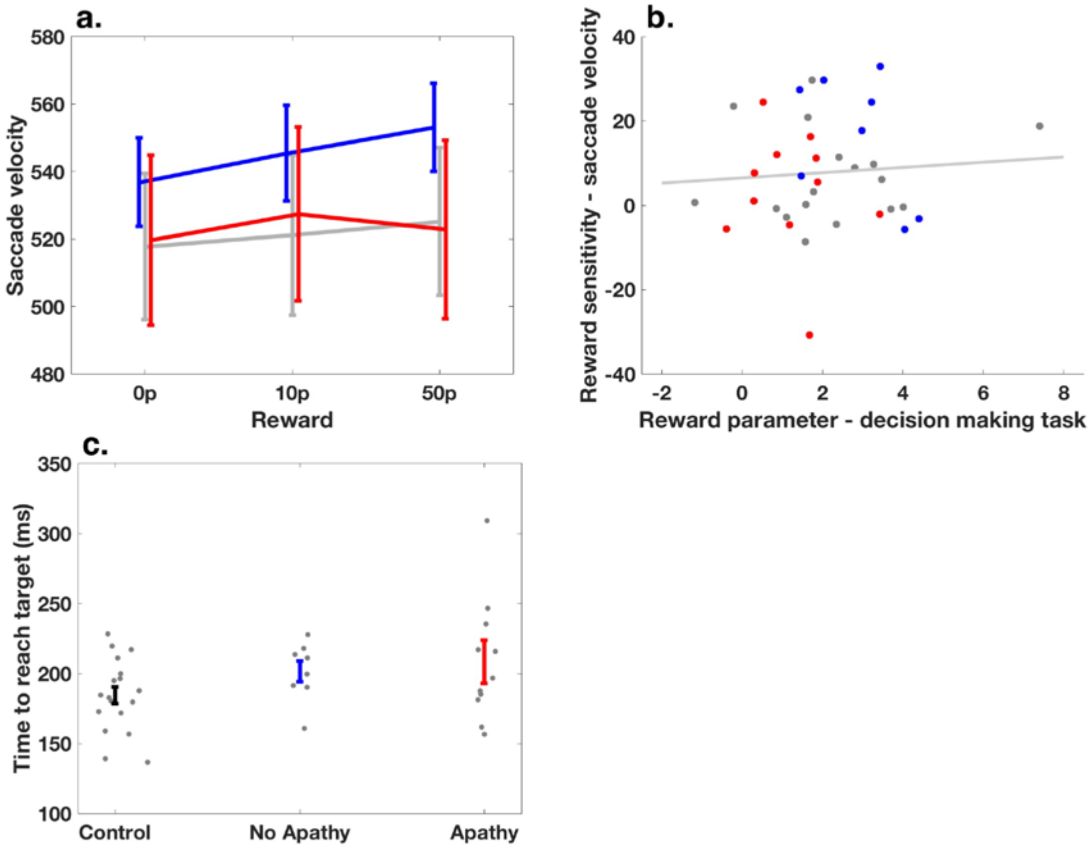

Supplementary figure 3.

There were no significant differences in peak saccade velocity between controls (grey), CADASIL no apathy (blue) or CADASIL apathy (red). There was a main effect of reward on velocity, with speed increasing with greater incentives: F(2,68)=6.8, p=0.002. There was no interaction between reward and group: F(4,68)=1.43, p=0.24, (**a**). Reward sensitivity, indexed by change in saccade velocity with increasing incentives, was not correlated with the parameter estimate for reward from the behavioural decision making task (**b**). Reaction time (time to saccade to target following cue to move) did not differ as a function of apathy (Mean Difference (No Apathy vs Apathy) = 7ms, t_{17}=0.41, p=0.69), nor was there a significant group effect: F(2,36) = 2.2, p=0.13 (**c**).

EFFECT OF DEPRESSION ON BEHAVIOURAL AND AUTONOMIC RESPONSES

Effort-based decision making task:

Repeated Measures ANOVA: We performed a repeated measures ANOVA to analyse how the proportional acceptance rate in each cell of the sampled 6x6 decision space varied with reward, effort and depression status. High depression (n=11) was defined as a score of ≥ 2 on the GDS-depression subscale, in line with previous work (Ligthart et al., 2012). GDS data was missing for one patient, who was therefore excluded from the analysis. There was no main effect of depression on acceptance rate (F(1,16)=2.2, p=0.16). Furthermore, there was no interaction between depression and either reward or effort:

Reward*Depression: F(1.6,25.4)=0.47, p=0.59
Effort*Depression: F(1.5,24.8)=0.65, p=0.49
Reward*Effort*Depression: F(5.3,85.2)=1.3, p=0.27

As previously, there were main effects of effort and reward and a two-way interaction between effort and reward (Effort: F(1.5,24.8)=18.8, p<0.001; Reward: F(1.6,25.4)=28.5, p<0.001; Effort*Reward: F(5.3,85.2)=3.7, p=0.004.

Computational model: There was no significant difference between low and high depression groups in the parameter estimates from the computational model. That is, depression status was not associated with a significant change in the effect of reward (t_{15}=1.6, p=0.13) or effort (t_{15}=0.05, p=0.96) on willingness to accept offers, nor on the baseline tendency to accept (intercept, t_{15}=0.98, p=0.34).

Eye movement task:

Pupil analysis: We assessed whether depression in CADASIL was associated with blunted pupillary responses to reward, as measured by the proportion change in pupil size between high reward (50p) and low reward (0p) conditions (the same measure used for the apathy contrast). There was no significant effect of depression on this metric: mean difference in proportional change (Low Depression – High Depression) = 0.44±0.37, t_{16}=1.2, p=0.25.

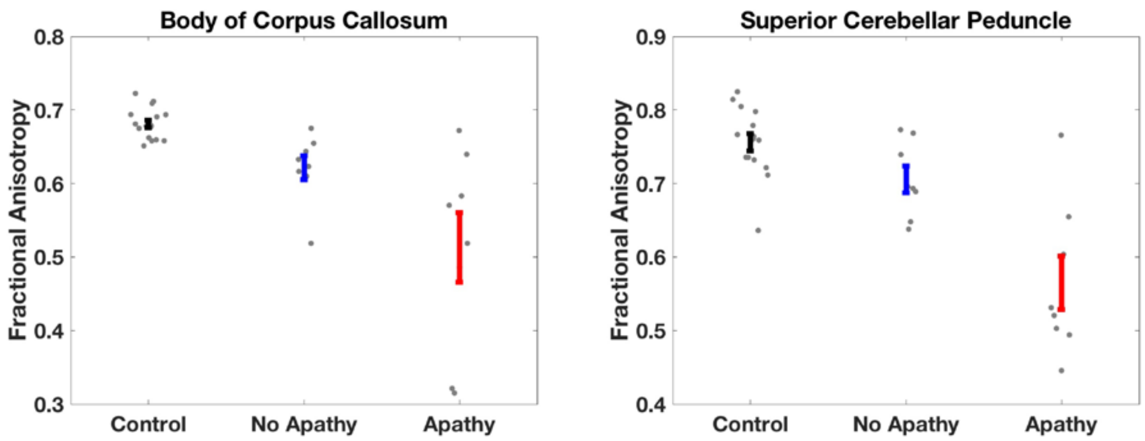

Supplementary figure 4.

Average FA values for controls, and non-apathetic and apathetic CADASIL patients, extracted from the significant areas in the apathy contrast.

RELATIONSHIP BETWEEN FA IN APATHY-ASSOCIATED REGIONS AND BEHAVIOURAL REWARD SENSITIVITY

We examined whether the FA values within apathy-associated regions were directly associated with the behavioural measure of reward sensitivity. Because these neural regions were defined by the contrast: apathy < no apathy, we orthogonalized the extracted FA values with respect to apathy (using each patient's LARS score). We used a general linear regression model, entering sequentially entering LARS score and then FA value (for each region) as predictor variables, and behavioural reward sensitivity as the outcome variable. FA values within the medial frontal white matter (underlying orbitofrontal and anterior cingulate cortex) and anterior limb of the internal capsule significantly improved model fit over a model just including LARS score:

OFC-ACC WM: $\Delta R^2 = 0.2$, F=5.3, p=0.04

ALIC: $\Delta R^2 = 0.3$, F=5.8, p=0.03

FA values within the anterior cingulum and body of the corpus callosum were not associated with behavioural reward sensitivity, after controlling for apathy:

AC: $\Delta R^2 = 0.16$, F=2.6, p=0.13

CCb: $\Delta R^2 = 0.07$, F=1.1, p=0.32

REFERENCES

Andersson, J.L.R., Jenkinson, M., Smith, S., Andersson, J., 2007. Non-linear registration aka Spatial normalisation FMRIB Technial Report TR07JA2.

Andersson, J.L.R., Skare, S., Ashburner, J., 2003. How to correct susceptibility distortions in spin-echo echo-planar images: application to diffusion tensor imaging. Neuroimage 20, 870–888. doi:10.1016/S1053-8119(03)00336-7

Andersson, J.L.R., Sotiropoulos, S.N., 2016. An integrated approach to correction for off-resonance effects and subject movement in diffusion MR imaging. Neuroimage 125, 1063–1078. doi:10.1016/J.NEUROIMAGE.2015.10.019

Chong, T.T.-J., Apps, M., Giehl, K., Sillence, A., Grima, L.L., Husain, M., 2017. Neurocomputational mechanisms underlying subjective valuation of effort costs. PLOS Biol. 15, e1002598. doi:10.1371/journal.pbio.1002598

Muhammed, K., Manohar, S., Ben Yehuda, M., Chong, T.T.-J., Tofaris, G., Lennox, G., Bogdanovic, M., Hu, M., Husain, M., 2016. Reward sensitivity deficits modulated by dopamine are associated with apathy in Parkinson's disease. Brain. doi:10.1093/brain/aww188

Prevost, C., Pessiglione, M., Metereau, E., Clery-Melin, M.L., Dreher, J.C., 2010. Separate Valuation Subsystems for Delay and Effort Decision Costs. J. Neurosci. 30, 14080–14090.

Smith, S.M., Jenkinson, M., Johansen-Berg, H., Rueckert, D., Nichols, T.E., Mackay, C.E., Watkins, K.E., Ciccarelli, O., Cader, M.Z., Matthews, P.M., Behrens, T.E.J., 2006. Tract-based spatial statistics: Voxelwise analysis of multi-subject diffusion data. Neuroimage 31, 1487–1505. doi:10.1016/j.neuroimage.2006.02.024

Smith, S.M., Jenkinson, M., Woolrich, M.W., Beckmann, C.F., Behrens, T.E.J., Johansen-Berg, H., Bannister, P.R., De Luca, M., Drobnjak, I., Flitney, D.E., Niazy, R.K., Saunders, J., Vickers, J., Zhang, Y., De Stefano, N., Brady, J.M., Matthews, P.M., 2004. Advances in functional and structural MR image analysis and implementation as FSL. Neuroimage 23, S208–S219. doi:10.1016/j.neuroimage.2004.07.051

Winkler, A.M., Ridgway, G.R., Webster, M.A., Smith, S.M., Nichols, T.E., 2014. Permutation inference for the general linear model. Neuroimage 92, 381–397. doi:10.1016/j.neuroimage.2014.01.060
